# Is ApoE ɛ 4 a good biomarker for amyloid pathology in late onset Alzheimer’s disease?

**DOI:** 10.1186/s40035-016-0067-z

**Published:** 2016-11-16

**Authors:** Maowen Ba, Min Kong, Xiaofeng Li, Kok Pin Ng, Pedro Rosa-Neto, Serge Gauthier

**Affiliations:** 1 Department of Neurology, Yuhuangding Hospital Affiliated to Qingdao Medical University, Qingdao, Shandong 264000 People’s Republic of China; 2 McGill Centre for Studies in Aging, McGill University, Douglas Institute, 6825 Lasalle Boul, Montreal, QC H4H 1R3 Canada; 3 Department of Neurology, Yantaishan Hospital, Yantai City, Shandong 264000 People’s Republic of China; 4 Department of Neurology, The Second Affiliated Hospital of Chongqing Medical University, Chongqing, 400010 People’s Republic of China; 5 Department of Neurology, National Neuroscience Institute Singapore, Singapore, Singapore

**Keywords:** Apolipoprotein E ɛ4, Alzheimer’s disease, Amyloid

## Abstract

Amyloid plaques are pathological hallmarks of Alzheimer’s Disease (AD) and biomarkers such as cerebrospinal fluid (CSF) β-amyloid 1–42 (Aβ1-42) and amyloid positron emission tomographic (PET) imaging are important in diagnosing amyloid pathology in vivo. ɛ4 allele of the Apolipoprotein E gene (ApoE ɛ 4), which is a major genetic risk factor for late onset AD, is an important genetic biomarker for AD pathophysiology. It has been shown that ApoE ɛ 4 is involved in Aβ deposition and formation of amyloid plaques. Studies have suggested the utility of peripheral blood ApoE ɛ 4 in AD diagnosis and risk assessment. However it is still a matter of debate whether ApoE ɛ 4 status would improve prediction of amyloid pathology and represent a cost-effective alternative to amyloid PET or CSF Aβ in resource-limited settings in late onset AD. Recent research suggest that the mean prevalence of PET amyloid-positivity is 95% in ApoE ɛ 4-positive AD patients. This short review aims to provide an updated information on the relationship between ApoE ɛ 4 and amyloid biomarkers.

## Background

Alzheimer’s disease (AD) is the most common neurodegenerative dementia, which severely impacts daily living. The medical cost for AD patients is also significantly high [1]. Advance in medical research have led to the discovery of biomarkers for the diagnosis of AD pathologies, such as decreased cerebrospinal fluid (CSF) β-amyloid 1–42 (Aβ1-42), positive amyloid positron emission tomographic (PET) imaging and presence of the Apolipoprotein E ɛ4 allele (ApoE ɛ 4) for amyloid pathology [2–6]. Aβ plaque is one of the main hallmarks of AD which is related to neuronal death [1, 7, 8]. CSF-Aβ1-42 and amyloid PET imaging are able to quantify the level of Aβ pathology while amyloid PET is able to show the distribution of Aβ deposits in the brain. However, the invasive examination of lumbar puncture and expensive tests of amyloid PET have restricted their use in clinical practice for AD diagnosis and risk assessment. The search for a cost effective biomarker with good prediction for AD pathology is the goal. ApoE ɛ 4 is one of the major and best-established genetic risk factor for late onset AD [9–11]. It has been shown that ApoE ɛ 4 is involved in Aβ deposition and the formation of amyloid plaques, which accounts for its role on the pathophysiology of AD and hence a potential biomarker for diagnosing amyloid pathology. Indeed, the development in neuroimaging technology has allowed us to assess the relationship between the ApoE ɛ 4 and amyloid PET imaging. This review aims to summarize the current evidences regarding the relationship between the ApoE ɛ 4 and amyloid biomarkers.

### Back to basic: the effect of ApoE ɛ 4 on Aβ

ApoE ɛ 4, which is positive in > 40% AD cases, is one of the strongest genetic risk factor for AD among the three human ApoE isoforms (ε2, ε3 and ε4 allele) [12, 13]. Histopathological studies of AD brains show that ApoE ɛ 4 coexist with Aβ in amyloid plaques [14], demonstrating an association between ApoE ɛ 4 and Aβ in the pathological structure of AD. Epitope analysis shows that the 144–148 residues in the N-terminal region of ApoE ɛ 4 and the 13–17 residues in Aβ as the receptor-binding domain [15], are common sites that interact with each other. ApoE ɛ 4 plays a key role in AD pathophysiology because it is less effective in breaking down Aβ peptide compared to other ApoE isoforms, which results in an increased risk of formation of amyloid plaques. Meanwhile, ApoE ɛ 4-containing lipoprotein is seldom lipidated, which reduces its stability and this leads to a lower level of ApoE ɛ 4/Aβ complex. The decreased level of ApoE ɛ 4/Aβ complex further leads to the increased Aβ aggregation. Several in vivo studies have also clearly shown that when ApoE ɛ 4 deficient mice crosses with APP transgenic mice, there is decreased Aβ deposition compared to human ApoE ɛ 2 and ApoE ɛ 3 [13, 16]. On the other hand, human ApoE ɛ 4 overexpression increases Aβ deposition [16–19]. A further detailed quantitative research of Aβ homeostasis using in vivo microdialysis in human ApoE deficient and human amyloid precursor protein crossed mice showed that Aβ clearance reduced the most in mice with by ɛ 4 allele, followed by ɛ 3 and then ɛ 2 alleles. [20]. These findings clearly show the significant role of ApoE ɛ 4 in the formation of fibrillar Aβ [16, 19] which results in cognitive impairment.

### Current in clinic: the association between ApoE ɛ 4 and Aβ

Low CSF Aβ1-42 and high amyloid PET imaging in the brain are biomarkers which may support the diagnosis of AD. With the advancement of amyloid PET imaging and amyloid ligands development, it is now possible to visualize amyloid plaques in vivo in the brain. As mentioned above, ApoE ɛ 4 is a major genetic risk factor for amyloid pathology in late onset AD [9–11]. The presence of one copy of the ApoE ɛ 4 allele increases the risk of late onset AD by about 3.7 times while the presence of two copies increases this risk by about 12 times as compared to the ApoE ɛ 3 isoform [21]. More importantly when compared with non-carriers, Aβ deposition and amyloid plaque formation is greater in ApoE ɛ 4 carriers. In the brain of ApoE ε4/ε4 AD patient, the level of Aβ oligomers is 2.7 times higher than ApoE ε3/ε3 AD patient and this corresponds to greater total amyloid plaque burden. This suggests that ApoE ɛ 4 influences Aβ oligomers metabolism. ApoE increases Aβ oligomers levels in an isoform dependent manner (ɛ 2 < ɛ 3 < ɛ 4) [22]. A report from the Alzheimer’s Disease Neuroimaging Initiative (ADNI) database shows the influence of ApoE ɛ 4 dose on clinical and neuroimaging biomarkers across the AD spectrum (from cognitive normal to AD patients with severe cognitive impairment. ApoE ɛ 4 is associated with decreased CSF beta-amyloid (Aβ_1–42_) and increased cerebral Aβ deposition across the AD spectrum. ApoE ɛ 4 increases cerebral amyloid-β (Aβ) deposition in all the stages of AD development, and also influences Aβ-initiated cascade of downstream neurodegenerative effects, thereby increasing the risk of AD [2]. A recent meta-analysis also shows that ApoE ɛ 4 carriers (either 1 or both alleles) were significantly associated with increased amyloid PET deposition, suggesting its potential effects on cortical amyloid burden [23]. The difference of amyloid plaque burden between ApoE ɛ 4 carriers and ApoE ɛ 4 non carriers patients, may be explained that Aβ deposition starts earlier and continues for a longer time in ApoE ɛ 4 carriers. This theory is supported by the research in neuropathology and epidemiology, which showed earlier onset of disease and higher amyloid plaque burden in younger ApoE ɛ 4 carriers with AD. Another possible explaination for greater plaque burden in ApoE ɛ 4 carrier is that there may be a higher speed of amyloid deposition in ApoE ɛ 4 carriers over time in the process of disease [24–27]. Further studies are needed to investigate this relationship, including subgroups analyses according to diagnosis from a more homogeneous population. In contrary, one study showed the increased amyloid deposition in the frontal cortex in ApoE ɛ 4 noncarriers [28]. It appears contradictory that lack of the important genetic risk factor for AD is related to increased amyloid burden. It was explained that the inconsistent outcome could be associated with confounding factors interfering with demographic characteristics, different assay protocols and even the accuracy of clinical diagnosis.

Indeed, there is a very high concordance between amyloid PET and CSF Aβ in AD patients, as demonstrated in the research on Pittsburgh compound B imaging and cerebrospinal fluid amyloid-β in a multicentre European memory clinic study published in Brain [29]. Yet, clinicians are more concerned whether the knowledge of ApoE ɛ 4 status would improve prediction of amyloid status and represent a cost-effective alternative to amyloid PET or CSF Aβ in resource-limited settings. One recent meta-analysis published in JAMA [30], which pooled the results of 29 cohorts worldwide to assess the prevalence of PET amyloid-positivity of different dementia syndromes as a function of age and ApoE ɛ 4 status. With a total sample of 1359 patients with a clinical diagnosis of probable AD, the curves of PET amyloid-positivity were formed based on age and ApoE ɛ 4 status. The prevalence of PET amyloid-positivity is higher in clinically diagnosed AD patients especially when ApoE ɛ 4-positive. The mean prevalence of PET amyloid-positivity is 95% in ApoE ɛ 4-positive AD patients. The prevalence of PET amyloid-positivity is always above 90% from age 50 to age 90 in clinically diagnosed ApoE ɛ 4-positive AD potients. The data presented good concordance between ApoE ɛ 4 and PET amyloid-positivity. However, in ApoE ɛ 4-negative AD patients, the prevalence of PET amyloid-positivity decreased with age from 86% at age 50 to 68% at age 90. The mean prevalence of PET amyloid-positivity is 77% in ApoE ɛ 4-negative AD patients. Although, the prevalence of amyloid-positivity is lower in older patients with clinically diagnosed AD, especially when ApoE ɛ 4-negative, which instead indicate that knowledge of ApoE ɛ 4 status would improve the positive predictive value of amyloid PET results in older patients with clinically diagnosed AD. The weakness of the research is that ApoE ɛ 4 status is dichotomized as either positive or negative, without regards to the dose of ApoE ɛ 4 alleles. The data from solanezumab phase 3 clinical trials clearly demonstrated that the dose of ApoE ɛ 4 alleles correlates with amyloid burden and diagnosis of AD [31]. The prevalence of PET amyloid-positivity is 98% in ApoE ɛ 4/4-positive AD patients. One table adapted from the Degenhardt publication summarized the information that the dose of ApoE ɛ 4 alleles is a good predictor of amyloid positivity (Table 1). ApoE ɛ 4 status are crucial factors when ordering clinical amyloid PET scans, especially in resource-limited settings.

**Table 1:** Three hundred seventy Subjects with clinical diagnosis of mild to moderate AD and known ApoE ɛ 4 genotype (adapted from the Degenhardt publication in Psychosomatics in 2016)

	Amyloid FBP PET positive*	Amyloid FBP PET negative*	Totals	Accuracy of clinical diagnosis
ApoE ɛ 4(−)	107	65	172	62%
ApoE ɛ 4(+/−)	133	18	151	88%
ApoE ɛ 4(+)	46	1	47	98%

Although, ApoE ɛ 4 genotype is associated with decreased CSF Aβ_1–42_ in AD patients [2, 32–34]. There were no enough available reports to assess the prevalence of concordance of ApoE ɛ 4 and CSF Aβ_1–42_-positivity in AD patients. Future research is still required to clarify the concordance of ApoE ɛ 4, CSF Aβ_1–42_ and amyloid PET positivity in AD patients. When the concordance is clarified, thus blood ApoE ɛ 4 genotype biomarker as one economic testing can be helpful in AD patients when considering amyloid evaluation in clinical practice, especially when an anti-amyloid drug would be available.

## Conclusions

In summary, these basic and clinic researches support that ApoE ɛ 4 is highly associated with amyloid pathology in the brain. Especially, in confirmed AD patients with ApoE ɛ 4+, ApoE ɛ 4 genotype positivity almost equals brain amyloid positivity from a qualitative point of view. Future research exploring the dose-effect association between ApoE ɛ 4 genotypes and amyloid neuropathology of AD, or in conjunction with other markers can help to better understand the pathophysiological role of ApoE ɛ 4 and improve the diagnostic accuracy in AD. Considering the above relationship, blood ApoE ɛ 4 genotype positivity is an important referred biomarker for amyloid pathology and should be considered for use in AD diagnosis and future pre-treatment biological testing when an anti-amyloid drug will be available.
